# Out-Patient Statistics and Cost

**Published:** 1913-12-20

**Authors:** 


### OUT-PATIENT STATISTICS AND COST.

### Supplementary Memorandum Issued by the Hospital Funds.*

As hospital workers and readers of the economics section of The Hospital are aware, one of the chief features of the revision of the uniform system of hospital accounts, brought about by King Edward's Hospital Fund, is the separation of expenditure on in- and out-patients under a more scientific system than that which was previously employed.

Formerly the cost per occupied bed was calculated after a certain sum for each out-patient (estimated by the individual hospital accountant) had been deducted from the total ordinary expenditure. Later one of the central funds, for the purposes of preparing statements of expenditure for guidance in making grants, named the unit figure to be used in each case in the out-patient calculation. Then came the regulations of the revised system, which indicated the lines on which an accountant should base his estimate. The issue of the present Memorandum indicates that further light on the subject has been found necessary. The circular is the outcome of representations to the three funds that the comparison of the cost per out-patient attendance at different hospitals is materially affected by variations in the method of keeping the statistics of in- and out-patients or in apportioning expenditure. As a result of these representations a committee to further consider the matter was formed, consisting of representatives of the central funds and seven hospital secretaries.

Answers to questions submitted to the various hospitals revealed certain differences of practice both in the enumeration of out-patients and their attendances and in the method of separating the total expenditure on out-patients from that on in-patients; these differences being so important as to detrimentally affect the value of the statistics published by the hospitals and summarised by the King's Fund.

As the proper registration of patients must be the basis of all average calculations, certain supplementary regulations, as a result of the deliberations of the committee, have been issued by the funds in respect of this important duty. In a department which is, or should be, managed by trained officers, and where estimates need never be employed as an element in a calculation, it must be painful to the general body of hospital managers to find that it has been found necessary to include the following regulations:—

"A register of new out-patients should always be kept, giving dates and stating the names and addresses of the patients."

"Attendances should not be reckoned by multiplying the number of patients by any figure representing the average attendances per patient."

"No additions to the count of attendances should be made in respect of the number of weeks' medicine given at any attendance."

The proper carrying out of most kinds of hospital duties is dependent upon the appointment of trained officers to managerial posts; subject to this, any uncertainty as to the right method of enumerating out-patients and attendances should now be removed.

The same certainty, we are sorry to say, cannot be felt in respect of the separation of the expenditure on in-patients from that on out-patients. The funds, according to the Memorandum, still think it desirable to leave responsibility as to separation to the various hospitals, and now intervene not to make definite rules, but to suggest general considerations for guidance in apportionment.

There are, of course, a few items which can be allocated direct to the expenditure of each side—certain salaries, a few surgery and dispensary charges, and the like. Some hospitals (for example, Hampstead General Hospital, with its out-patient department in another building) can separate cost with a near approach to accuracy. But in no case can the division be made without some use of personal judgment in apportionment by the responsible officer. The Memorandum says: "Such apportionments, as is well known to all men of business, are regularly made in manufacturing concerns, where a competitive business is done, and the cost of various goods manufactured on the premises has to be arrived at to enable contracts to be dealt with and the profit and loss thereon to be ascertained."

But one can find no analogy which is of service in considering the present issue between a manufacturing concern and a hospital. If one department of a business err in calculating the cost of an article the house as a whole bears the consequences. Should a hospital overestimate its out-patient cost it gains an unfair advantage over other hospitals. Most of the people who look at the figures are not statisticians; the difference of, say, 7d. and 4d. in the cost of an out-patient attendance appears to them a trifling thing. Yet in a hospital of 200 beds and 40,000 out-patient attendances, to use the higher figure, instead of the lower, results in bringing down the cost of the occupied bed by £2 10s. After all, the cost per occupied bed represents the main outlay, and is the criterion used by the public in considering economy. Perhaps by this time natural evolution in hospital affairs has led us to assessment in this matter by a disinterested body. Such a process was not some years ago the same thing it is to-day. Now it is possible to base calculations on figures prepared on scientific lines for ten consecutive years. The Central Funds should be able, bearing in mind the type, size, and unusual conditions of an institution, to name a fair unit in each case to represent the expenditure on an out-patient attendance.

The synopsis of index of classification has been further revised and augmented, and a new heading is added to the receipts side of the income and expenditure account to take in payments under the National Insurance Act, whether in respect of individual insured patients or otherwise.

The Memorandum does not appear to give any direction as to from what date the new regulations shall be applied, but it is presumed that they cannot refer to the current year.

* Published by Geo. Barber, 23 Furnival Street, Holborn, E.C. Price 6d. post free.

